# A meta-analysis of depressive symptoms among Ethiopian prisoners and a narrative description of its associated factors: a country based systematic review and meta-analysis study

**DOI:** 10.1186/s12888-020-02662-5

**Published:** 2020-06-05

**Authors:** Mogesie Necho, Asmare Belete, Mekonnen Tsehay, Yosef Zenebe

**Affiliations:** Department of Psychiatry, Wollo University, College of Medicine and Health Sciences, Dessie, Ethiopia

**Keywords:** Meta-analysis, Depression, Ethiopia, Prisoners

## Abstract

**Background:**

The burden of depression in prisoners is increasing and factors such as co-existence of medical illness, lack of social support and longer duration of sentences are contributing to it. However, no pooled evidence on the magnitude and factors of depression in prisoners existed in Ethiopia. The current meta-analysis was therefore aimed to have aggregate evidence on the magnitude and factors of depression in prisoners of Ethiopia.

**Methods:**

A search of databases on PubMed, Scopus, and EMBASE was carried out systematically. Besides, grey literature sources were extensively investigated. Moreover, the reference lists of the articles selected were searched. Random effects and quality-effects models were used to describe the pooled prevalence of depressive symptoms with 95% CI. We also detect heterogeneity between studies using Cochran’s Q- statistic and the Higgs I^2^ test. A sensitivity analysis was also implemented. Publication bias was checked with Egger’s test and funnel plots visually.

**Results:**

Among 232 papers identified through the specified database searches only 17 full-text articles were assessed for eligibility and only nine (9) studies fulfilled the prespecified criteria and incorporated in the final meta-analysis. The pooled prevalence of Depressive symptoms among prisoners was 53.40%(95% CI: 41.33, 65.46). The pooled prevalence of Depressive symptoms in prisoners was 41.9% in Southwest Ethiopia, 44.43% in North West of Ethiopia, 59.05% in Addis Ababa, and 72.7% Southern Ethiopia. Besides, the pooled Depression symptoms prevalence among prisoners was 51.24% as measured with PHQ-9 and 56.15% with BDI-II. Besides, studies that utilized a relatively large sample size (≥350) yields a smaller pooled prevalence of Depression symptoms, 51.93% than those which utilized smaller sample sizes (< 350); 54.13%.

**Conclusion:**

The pooled magnitude of depression in prisoner’s population is very high, 53.40%. This pooled effect size for the Depression symptoms was significantly higher in the southern region of the country than in the southwest region. Besides, the pooled prevalence was significantly higher as measured by the BDI-II tool than by PHQ-9. Also, studies that utilized a larger sample size provided a significantly lower pooled magnitude of symptoms of depression than studies that utilized a smaller sample size.

## Background

Ethiopia has huge number of prisons both at the federal and regional levels. Three of the Ethiopian prisons namely, Kality prison, Diredawa prison, and Shewarobit prison are under the administration of the federal government. Mostly the federal prisons are used basically to handle serious crimes. Kality Prison; synonymously called Kilinto Prison is among the federal prisons and located in Addis Ababa and serves as the main prison of the country where the most severe form of crime that needs a long duration of sentences is handled there. Besides, around 9 regional prisons are found in the 9 regions of the country [1]. Normally prisoners in Ethiopia are involved in skill-building activities while they are in prison which is essential for their future life as they are mostly in the productive age group.

In 2001 World Health Organization (WHO) projected that depression will account for the second leading burden of diseases globally in 2020 [2]. More than 66 million people are affected by depression in the world as reported from the world health organization in which 85% reside in countries with low and middle income [3]. Mental illness is also highly prevalent in correctional institutions [4] with the magnitude of severe psychiatric problems up to 5–10 times higher among prisoners as compared to the general population [5]. WHO reported that 11% of world-wide prisoners were affected by mental health problems [6]. In prisons from Europe, the magnitude of psychotic disorders was 5%, depression and anxiety disorders were 25%, and disorders of substance use was approximately 40% [7].

A systematic review and meta-analysis of 62 studies in 12 countries showed that 3.7 and 65% of male inmates were with psychotic illnesses and a personality disorder respectively whereas 4 and 42% of female prisoners had a psychotic illness and a personality disorder respectively [8]. Another meta-analysis on 109 studies from 24 countries and a total of 33,588 inmates revealed that the pooled magnitude of psychosis was 3.6% in male inmates and 3.9% in female inmates [8]. In Ethiopia, too mental illness is common in prisoners with anxiety to be 36.1% in Amhara [9], suicidal behavior’s 23.2% in Jimma [10] and common mental disorders 58.4% in Addis Ababa [11] and substance use disorders,55.9% [12].

Depression is prevalent in prisoners [13]. A review and meta-analysis study in Iran on a total of 1708 prisoners and 12 articles obtained a pooled magnitude of depression to be 42%; 44% of male prisoners and 33% of female prisoners [14]. Another meta-analysis reports indicated that 10% of male and 12% of female prisoners [8] and 10.2% of male and 14.1% of female prisoners [8] had depression. Other preceding studies publicized that depression in prisoners was 24% in France [5], 19.2% in Norway [15], 40.7% in Malaysia [16], 8.1% in Chile [17], 85% in Pakistan [18], and 35% of convicted inmates and 30.1% of prisoners awaiting trial in Nigeria [19]. Studies on prisoners of Ethiopia showed that the magnitude of depression in prisoner’s population ranges between 41.9 and 89% [10, 20–26] higher than the pooled estimate of depression obtained by a meta-analysis study among the general population; 11% [27]. Regional distribution of depression in Ethiopian prisoners can be described as ranging from 43.8 to 45.5% in Amhara region [20, 22, 25], 56.4 to 89% in southern Ethiopia [21, 24], 51.6 to 66.5% in Addis Ababa [26, 28] and 41.9% in Oromia region [10, 23].

A national health survey on depression in Ethiopia identified 9.1% of the population to be affected by depression [29]. Moreover, A review and meta-analysis of depression in the Ethiopian community found a pooled depression prevalence of 20.5% [30] which was relatively high and implies a huge public health concern of the problem. However, only a few people with depression were found to have access to treatment and this high unmet need for depression might also contribute to criminal involvement.

A review and meta-analysis of 14 studies identified multiple modifiable factors (environmental, family support and psychological factors) as well as non-modifiable factors (social, demographic, individual and biological factors) as contributing to depression in prisoners [31]. Factors contributing to a variation of prevalence between studies in prisoners were gender, instruments used and offense categories (violent offenses) [14]. Identified determinants for depression in prisoners were female sex [8, 32–36], old age [37, 38], multiple incarcerations and work burden inside the prison [8, 39, 40] and history of mental illness [40, 41] from countries abroad whereas in the context of Ethiopia co-morbid medical illness [21, 23, 25, 26], longer duration of a sentence [20, 25, 26], poor social support [22, 23], family history of mental illness, previous incarceration and lifetime alcohol use [23], a suicidal attempt and ever-faced a stressful event [25] were mentioned so far as associated with depression in a correctional setting.

Depression is accounted for as the burden of the third most common disease in Ethiopia [42]. It affects multiple domains of life such as increased involvement in substance use [43], suicide risk [44–46], elevated risk for diseases of metabolic syndrome [47, 48] and advances in the occurrence of other medical illnesses [49–51]. Moreover, impairment in interpersonal and occupational life [42, 44, 47], and early mortality and morbidity, as well as the impaired country economy, would happen [30, 52, 53] due to depression.

Pieces of the literature revealed that most of the world prisoners resides at low- and middle-income countries (LMICs) [15] including Ethiopia However, there existed a huge unmet need for treatment of mental illness in general [54–56] and depression in particular [57, 58] in prisoners and the general population as well. Such a gap in treatment for depression in Ethiopia should be addressed scientifically by supplementing concrete evidence about the burden of depression for policymakers and concerned stakeholders which would be helpful for sound decision making. Nevertheless, no previous systematic review and meta-analysis studies investigated the magnitude of depression in prisoner’s population in Ethiopia. Therefore current systematic review and meta-analysis study aims to have a summary of pooled evidence in Ethiopia on the magnitude of depression in prisoner population and associated factors of depression in prisoner population as well as providing a recommendation for future researchers and other stakeholders.

## Methods

The current systematic review and meta-analysis were adherent to the Preferred Reporting Items for Systematic Reviews and Meta-analysis Protocols (PRISMA-P) [59]. The PRISMA checklist is found in Additional file 1: PRISMA 2015 checklist.

### Data sources and search strategies

A systematized search of PubMed with no time boundary, EMBASE, and Scopus databases were implemented under a detailed search strategy. We conducted our search in PubMed with the following key terms and words: (Prevalence OR epidemiology OR magnitude OR incidence) AND (depressive OR “depressive disorder” OR “depressive symptoms”) AND (prisoner’s OR inmates OR detainees OR incarcerated) AND (factor OR risk OR “risk factor” OR determinant) AND Ethiopia.

We used the conjunction “AND” to join the key terms above since our primary objective was to obtain studies that had assessed both depressive symptoms and the associated factors for depressive symptoms. Subject-specific heading as recommended by database searches was used for Scopus and EMBASE searches. Furthermore, unpublished articles on WHO websites, non-indexed articles from Google scholar were investigated. Moreover, the reference lists of included studies were manually searched for additional eligible articles. We also contacted authors of the merged articles when further information was required.

### Inclusion criteria

We were pre-planned to include cohort, case-control and cross-sectional studies which reported the magnitude of depressive symptoms and/or its associated factors in prisoners’ population in Ethiopia. But, all of the studies obtained by stated search strategies were found to be a cross-sectional study. We designed and implemented on behalf of the following pre-specified inclusion criteria:

A. Cohort, case-control and cross-sectional studies.
B. Studies should be conducted on prisoners
C. Publication of articles should be in the English language
D. Articles that assessed Depression symptoms prevalence in prisoners
E. Articles that identified associated factors of depressive symptoms in prisoners
F. Studies must be conducted in Ethiopia

### Exclusion criteria

A. Meta-analysis studies, experimental studies, commentaries, letters, and editorials were excluded.
B. Duplication studies were also checked and removed before the pooling process started.

### Selection of studies for inclusion in the review

We authors of this review utilized the stated databases above to retrieve relevant articles that were stored for further management and use in an EndNote reference manager.

Three review authors (MN, AY, and MT) independently reviewed each study’s title and abstracts from the EndNote reference manager and disagreements between these authors regarding the inclusion process were addressed through discussion and the fourth reviewer (YZ).

### Data extraction and management techniques

A standardized form was used to extract data from the included studies; three review authors (MN, AB, and MT) independently extracted data from included studies. The following parameters were utilized as components of a data extraction form and each of the authors extracting data use these parameters uniformly. The last name of the first author, publication year, study setting, sample size, number of individuals with depression, the magnitude of depression, assessment instruments, associated factors, and odds ratio (OR) with 95% confidence intervals (CI) were parameters used in extracting data from included studies.

### Quality assessment methods

Two review authors (MN, YZ) independently assessed the quality of studies included in the analysis using the Newcastle-Ottawa Quality Assessment tool in its modified version [60]. Representativeness and size of the sample, comparability between study subjects, ascertainment of depressive symptoms, and statistical quality were the dimensions of the Newcastle-Ottawa scale in assessing the quality of each study. We take the average of two reviewers (MN and YZ) to determine the final quality score of that individual study and any disagreement between them was settled by a discussion with a third reviewer (AB).

### Data synthesis and analysis

The average magnitude of depression and the average OR of associated factors for depression with 95% CI was determined with random-effects [61] and quality-effects models [62]. We also employed Quality-effects meta-analysis to know how the quality of each study affects the average estimate compared with the results from random-effects meta-analysis. This analysis takes into consideration the quality score of individual studies in the estimation of the study weight.

Heterogeneity between the studies had been assessed with both Cochrane’s Q statistic and the Higgins I^2^ test statistics which showed the variance between the included studies that were not due to a sampling error. In this review, a value of I^2^ statistics greater than 50% was used to detect the presence of heterogeneity between studies [63]. Since heterogeneity existed in the study, subgroup analyses and sensitivity analysis were performed to explore heterogeneity sources. Meta-XL version 5.3 [64] and STATA11 meta-prop packages [65] were used in the analysis. Publication bias was also assessed with funnel plot [66] and eggers publication bias test.

## Results

### Identification of studies

**Fig. 1:**
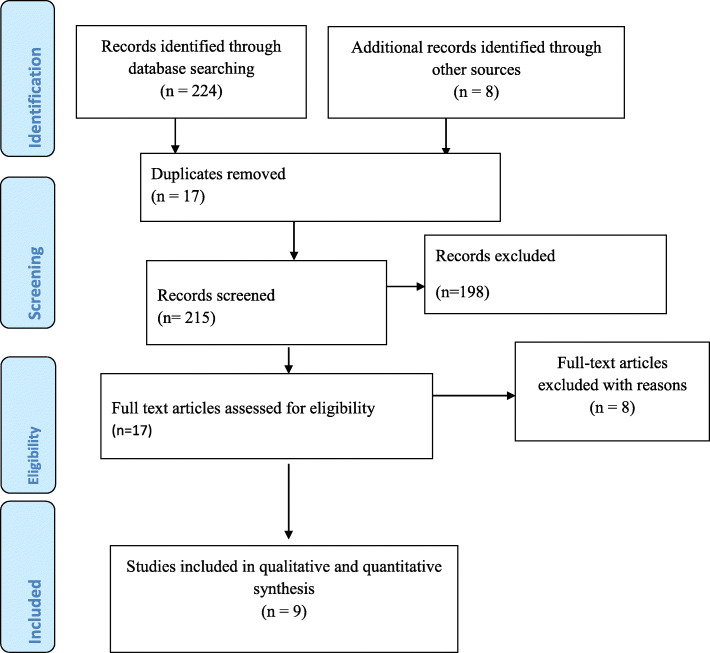
PRISMA flow chart for the review search process

Our search for both published work and grey literature resulted in 232 documented papers. Among these 8 were identified unpublished works and the remaining 224 were published papers notified from the search through previously stated database searches. Of these records, 17 were excluded since they were duplicates and further 198 articles were excluded simply by reading their titles. The remaining 17 studies were fully assessed for eligibility but only 9 studies were incorporated in the final meta-analysis since the rest 8 studies were also excluded because of varieties of methodological and technical limitations (Fig. 1).

### Characteristics of included studies

A total of 9 studies that assessed depressive symptoms in 3201 Ethiopian prisoners were incorporated into this review and meta-analysis study [10, 20–26, 28]. All included studies were institution based cross-sectional studies fulfilling the predefined inclusion criteria. One of these studies utilized a qualitative method (in-depth interview) of data gathering for associated factors of prisoner’s depressive symptoms in addition to the quantitative method [24]. Of these included studies, three were from Amhara region, Ethiopia [20, 22, 25], two other studies were from Oromia region, southwest Ethiopia [10, 23], two studies were from Addis Ababa, central Ethiopia [26, 28] and last two studies were also from the southern part of the country [21, 24]. Four of the nine studies included in meta-analysis utilized BDI-II [10, 23, 24, 28] for the assessment of depressive symptoms and the remaining five utilized PHQ-9 screening instruments [20–22, 25, 26]. Regarding the size of the sample utilized by the individual study three [20, 22, 26] of the nine used a sample size of greater than 350 and the rest six [10, 21, 23–25, 28] have a sample size less than 350.

The sampling technique used in four of the studies [20, 21, 25, 26] was simple random sampling whereas another four studies utilized a systematic sampling method in recruiting their study subjects [10, 23, 24, 28]. The last one used a multi-stage sampling technique [22] (Table 1).

**Table 1:** Characteristics of studies on depression in prisoners which are incorporated in the narrative as well as meta-analysis according to author first name, year of publication, setting of study, design, sample size, assessment instrument, study population and magnitude of depression

Author, year	Location of prison	Study design	Sample size	Tool (cutoff point)	Study population	Depression(%)(n)	Sampling Method
Alemayehu et al. 2019 [20]	Amhara, northwest Ethiopia	CS	402	PHQ-9(≥5)	Prisoners	45.5(*n* = 183)	Simple random
Reta et al. 2019 [25]	Amhara region	CS	336	PHQ-9(> 5)	Prisoners	44(*n* = 148)	Simple random
Beyen et al. 2017 [22]	Amhara, northwest Ethiopia	CS	649	PHQ-9	Prisoners	43.8(*n* = 284)	Multi-stage-random
Abdu et al. 2018 [23].	Oromia, south west Ethiopia	CS	332	BDI-II	Prisoners	41.9(*n* = 139)	Systematic sampling
Agegnew et al. 2019 [24]	Southern Ethiopia	CS	327	BDI-II	Prisoners	89(*n* = 291)	Systematic sampling
Bedaso et al. 2018 [21]	Southern Ethiopia	CS	335	PHQ-9(≥5)	Prisoners	56.4(*n* = 189)	Simple random
Getaneh et al. 2019 [26]	Addis Ababa, central Ethiopia	CS	400	PHQ-9(≥5)	HIV-positive prisoners	66.5(*n* = 266)	Simple random
Tefera, 2018 [28]	Addis Ababa, central Ethiopia	CS and qualitative	84	BDI-II	Women inmates	51.6(43)	Systematic sampling
Tirfeneh.et al. 2018 [10]	Oromia, south west Ethiopia	CS	336	BDI-II ≥ 14	prisoners	41.9(141)	Systematic sampling

Key: BDI II: Beck depression inventory II, CS: Cross-sectional, HIV: Human Immune-Virus, PHQ-9: Patient Health Questionnaire-9

### Quality of included studies

Generally, the overall score of quality assessment for the studies incorporated in the current meta-analysis varies from 6 to 10. Among the nine included studies, more than half (5 studies) had good quality and the rest 4 studies had moderate quality. None of the included studies were found to have poor quality (Additional file 2).

### The pooled prevalence of depressive symptoms in prisoners in Ethiopia

**Fig. 2:**
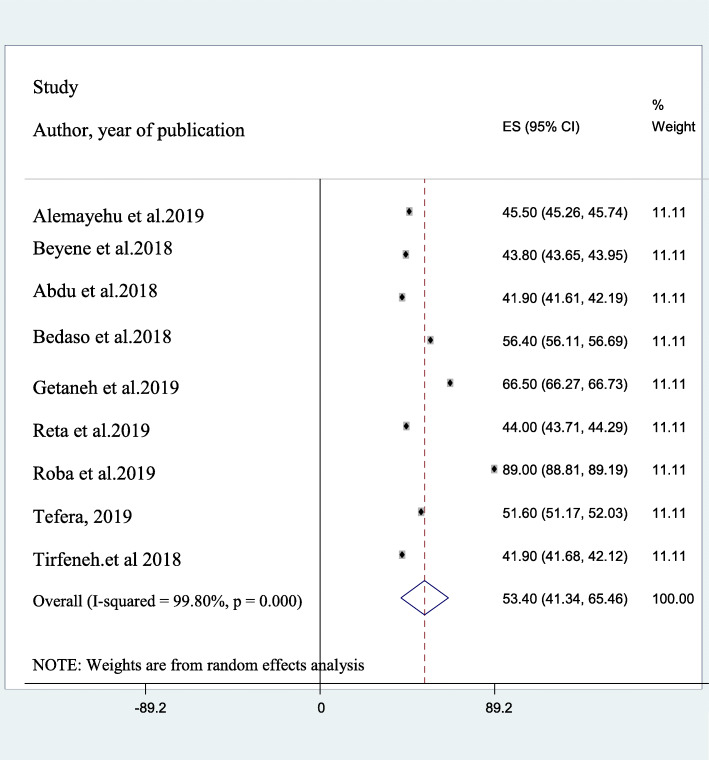
A forest plot for the prevalence of depression among prisoners in Ethiopia

Nine studies had reported the magnitude of depressive symptoms in prisoners in Ethiopia [10, 20–26, 28]. The reported magnitude of depressive symptoms among studies included in the current review and meta-analysis ranges from 41.9% [10, 23] in two studies from Oromia, southwest Ethiopia to 89% [24] in southern Ethiopia. The average prevalence of depressive symptoms among prisoners in Ethiopia using the random effect model ranges between 41.3 and 65.5%. This pooled magnitude was having a significant heterogeneity (I^2^ = 99.8%, *p*-value < 0.001) from the variation between the included studies (Fig. 2).

### Subgroup analysis of the prevalence of depressive symptoms in the prisoner’s population in Ethiopia

The result of a subgroup analysis based on the region from four areas of the country; Amhara region, [20, 22, 25], Oromia region [10, 23], Addis Ababa [26, 28] and Southern part of the country [21, 24] revealed that the pooled prevalence of Depressive symptoms in prisoners was 44.43, 41.9, 59.1, and 72.7% respectively. Besides, the subgroup analysis of Depressive symptoms in prisoners concerning the assessment tool used implied that the pooled depressive symptom prevalence among prisoners as measured with PHQ-9 was found to be 51.2% whereas it is 56.2 in those studies which assessed depression with BDI-II. Besides, studies that utilized a relatively large sample size (≥ 350) [20, 22, 26] yields a smaller pooled prevalence of depression, 51.93 than those studies which utilized smaller sample size (< 350) [10, 21, 23–25, 28] in which the average prevalence was 54.1 (Table 2).

**Table 2:** A subgroup analysis of the prevalence of depression among prisoners in Ethiopia based on random effect analysis

Subgroup		Number of studies	Estimates		Heterogeneity		
			Prevalence (%)	95% CI	I^2^	Q(DF)	*p*-value
Region	Amhara	3	44.43	43.36, 45.550	98.6%	139.17(2)	*P* < 0.001
	Oromia	2	41.9	41.73, 42.07	0%	0% (1)	*P* = 1.00
	Southern part	2	72.7	40.75, 104.65	99.8%	3420(1)	P < 0.001
	Addis Ababa	2	59.05	44.45, 73.65	99. 2%	1246.23(1)	P < 0.001
Tools	PHQ-9	5	51.24	42.23, 60.25	98.8%	921.57(4)	P < 0.001
	BDI-II	4	56.15	29.09, 83.11	99.6%	1742.86 (3)	P < 0.001
Sample	< 350	6	54.13	35.66, 72.60	99.2%	1242.12 (5)	P < 0.001
	≥ 350	3	51.93	38.08, 65.78	98.8%	935.75(2)	P < 0.001
Sampling technique	Simple random	4	53.1	42.23, 63.97	99%	1150.75(3)	P < 0.001
	Systematic and multi-stage	5	53.64	33.15, 74.13	99.4%	1624.13(4)	P < 0.001

Key: *BDI II* Beck depression inventory II, *DF* Degree of Freedom, *PHQ-9* Patient Health Questionnaire-9

### Sensitivity analysis

For further investigation of the source of heterogeneity in the analysis of the prevalence of depressive symptoms in prisoners, we performed a leave-one-out sensitivity analysis. Our sensitivity analysis result showed that the point estimated prevalence obtained when each study is left out from analysis was inside the confidence interval of the pooled depressive symptom prevalence. Therefore, the result of the pooled magnitude of depressive symptoms in prisoners can be plausible. The one study leave out at a time analysis showed that the pooled depressive symptoms prevalence ranges between 48.9 (42.69, 55.21) and 54.8 (41.72, 67.95) after the omission of a single study (Table 3).

**Table 3:** A sensitivity analysis of the prevalence of depression in Ethiopian prisoners when each indicated studies are removed at a time with its 95% confidence interval

No	Study excluded	Prevalence of depression	95% Confidence interval
1	Alemayehu et al. 2019 [20]	54.39	40.95, 67.83
2	Beyen et al. 2017 [22]	54.60	40.85, 68.35
3	Abdu et al. 2018 [23]	54.84	41.72, 67.95
4	Bedaso et al. 2018 [21]	53.03	39.63, 66.42
5	Getaneh et al. 2019 [26]	51.76	38.42,65.10
6	Reta et al. 2019 [25]	54.58	41.37, 67.78
7	Agegnew et al. 2019 [24]	48.95	42.69, 55.21
8	Tefera, 2018 [28]	53.63	40.56, 66.69
9	Tirfeneh, 2018 [10]	54.84	41.53, 68.15

### Publication Bias

**Fig. 3:**
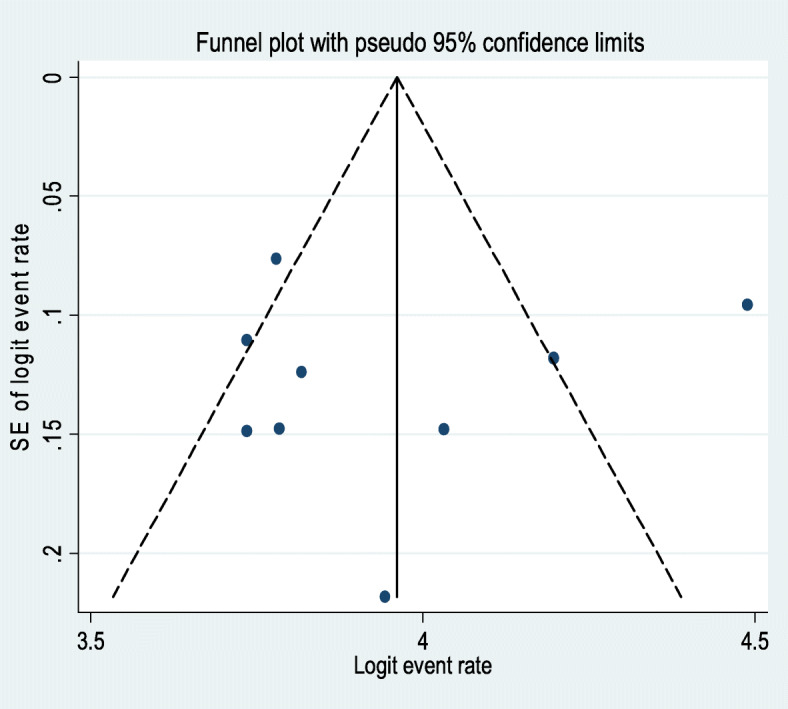
Funnel plot of the risk of publication bias for the prevalence of depression in prisoners

The egger’s publication bias plot is around the origin and Egger’s regression tests (*P* = 0.785) provided then no significant publication bias for the prevalence of depressive symptoms in prisoners in Ethiopia. Also, a funnel plot for a Logit event rate of prevalence of depressive symptoms in prisoners against its standard error strengthened this (Fig. 3).

### Factors associated with depression in Ethiopian prisoners

Among 9 studies included 7 studies that conveyed the factors associated with depressive symptoms in prisoner’s population in Ethiopia were incorporated in the qualitative analysis for associated factors of prisoner’s depressive symptoms [20–23, 25, 26, 28] (See Table 4). Association between comorbidity of medical illness in prisoners was a contributing factor for prisoner’s Depressive symptoms in four of the included studies [21, 23, 25, 26]. Besides, narrative review of the factors illustrated that longer duration of the sentence was found to have an association with the development of depressive symptoms in prison [20, 25, 26], poor social support was also noticed to be a contributing factor for depressive symptoms in two of the included studies [22, 23]. Moreover, a family history of mental illness, previous incarceration and lifetime alcohol use [23], a suicidal attempt and ever-faced stressful event [25] were some of the mentioned factors.

**Table 4:** Characteristics of associated factors for depression in prisoners in Ethiopia by their Odds ratio, Confidence interval, association strength, author and year of publication

Associated factors	Odds ratio(AOR)	95% CI	Strength of association	Author, year of publication
Having children	2.48	1.60,3.83	Moderate, positive	Alemayehu et al.,2019 [20]
Health satisfaction rated as moderate	3.2	1.12,9.00	Strong and positive	Alemayehu et al. 2019 [20]
Health satisfaction rated as dissatisfied	1.63	1.02,2.62	Moderate, positive	Alemayehu et al. 2019 [20]
Being sentenced for more than 5 years	2.31	1.01,5.25	Moderate, positive	Alemayehu et al. 2019 [20]
Being sentenced for 1–5 years	3.04	1.20,7.71	Strong and positive	Alemayehu et al. 2019 [20]
Not satisfied with day to day life before imprisonment	0.44	0.26,0.63	Moderate, negative	Beyen et al. 2017 [22]
Thought of facing difficulty to run life as before after release	1.87	1.3,2.69	Moderate, positive	Beyen et al. 2017 [22]
Having a plan to commit suicide	4.16	2.56,6.77	Strong and positive	Beyen et al. 2017 [22]
Good social support	0.62	0.44,0.89	Strong and negative	Beyen et al. 2017 [22]
Prison setting being at Gondar	1.54	1.04,2.29	Moderate, positive	Beyen et al. 2017 [22]
Prison setting being at Debre-tabor	2.27	1.46,3.51	Moderate, positive	Beyen et al. 2017 [22]
Having family history of mental illness	6.05	2.60,13.8	Strong and positive	Abdu et al. 2018 [23]
Having chronic physical illness	2.87	1.29,6.41	Moderate, positive	Abdu et al. 2018 [23]
Having history of previous incarceration	3.26	1.02,10.64	Strong and positive	Abdu et al. 2018 [23]
Lack of job in the prison	4.96	2.09,11.8	Strong and positive	Abdu et al. 2018 [23]
Lifetime alcohol use	3.61	1.80,7.26	Strong and positive	Abdu et al. 2018 [23]
Thinking life to be a difficult one after release from prison	2.07	1.20,3.60	Moderate, positive	Abdu et al. 2018 [23]
Having age between 21 and 25 years	2.04	1.06,3.89	Moderate, positive	Abdu et al. 2018 [23]
Poor social support	2.2	1.27,3.82	Moderate, positive	Abdu et al. 2018 [23]
Primary education	4.17	1.65,10.48	Strong and positive	Getaneh et al. 2019 [26]
Perceived stigma	3.88	2.08,7.25	Strong and positive	Getaneh et al. 2019 [26]
History of chronic illness	2.88	1.34,6.17	Moderate, positive	Getaneh et al. 2019 [26]
WHO clinical stage II AIDS	2.47	1.19,5.12	Moderate, positive	Getaneh et al. 2019 [26]
Length of stay in prison 4–6 years	2.27	1.22,4.23	Moderate, positive	Getaneh et al. 2019 [26]
Length of stay in prison ≥10 years	3.5	1.15,10.85	Strong and positive	Getaneh et al. 2019 [26]
Not participating in IGA inside the prison	0.53	0.32,0.87	Moderate, negative	Bedaso et al. 2018 [21]
Having chronic disease	2.62	1.29,5.32	Moderate, positive	Bedaso et al. 2018 [21]
History of Chat chewing	2.47	1.04,5.85	Moderate, positive	Bedaso et al. 2018 [21]
Widowed	6.3	1.09,36.67	Strong and positive	Reta et al. 2019 [25]
Educated at college or university level	5.34	1.59,17.94	Strong and positive	Reta et al. 2019 [25]
A history of suicidal attempt	2.76	1.04,7.31	Moderate, positive	Reta et al. 2019 [25]
Ever faced a severe stressful life event	2.57	1.41,4.67	Moderate, positive	Reta et al. 2019 [25]
5–10 years of sentence	2.5	1.32,4.79	Moderate, positive	Reta et al. 2019 [25]
Having chronic medical illness	3.32	1.26,8.75	Strong and positive	Reta et al. 2019 [25]

*IGA* Income Generating Activities

*WHO* World Health Organization

*AIDS* Acquired Immune Deficiency Syndrome

Considering the strength of association; health satisfaction rated as moderate, being sentenced for 1–5 years [20], having a plan to commit suicide [22], having a family history of mental illness, having a history of previous incarceration, lack of job in the prison, lifetime alcohol use [23], primary level of education and perceived stigma, long stay in prison ≥10 years [26], being widowed, educated at college or university level and having a chronic medical illness [25] had a strong and positive association with depression in Ethiopian prisoners (Table 4).

## Discussion

To the investigator’s knowledge, this review and meta-analysis on the prevalence of depression and the associated factors in individuals at the prison setting are the first of its type in Ethiopia in this target population. Therefore, the knowledge generated from this meta-analysis on pooled magnitude and risk factors for depression at prisoners of Ethiopia will be important evidence to different stakeholders intending to design policy in the area.

The result of the meta-analysis showed that depression in the prisoners in Ethiopia was high (53.40%). This high pooled result revealed that depression in prisoners is an important public health problem.

In the current review and meta-analysis, the existing available information varies by a region where the study was conducted, data collection instrument used to screen depression, the sample size incorporated in the study and sampling technique employed in data collection. More than half (*n* = 5) of the incorporated studies utilized PHQ-9 to screen depression whereas the rest utilized BDI-II. Two-third of the included studies utilized a sample size of less than 350.

In this meta-analysis, the pooled depression prevalence among prisoners in Ethiopia (53.4%) was Larger than an Iranian review and meta-analysis study on 1708 prisoners and 12 articles in which a pooled magnitude of depression was 42% [14] and also another meta-analysis that reported 10% of males and 12% of female worldwide prisoners as having depression [8]. The reason for such a high magnitude in the Ethiopian context would be due to the poor setting of prison and poor health care coverage, poor fulfillment of basic human needs. Moreover, differences in administrative procedures in the prison setting and variation in constitutional affairs across such countries could bring the discrepancy. Besides, variation in socio-cultural and environmental factors should be taken into consideration.

Besides, this result was also bigger than a national-level estimate of depression in Ethiopia which was reported to be from 9.1 to 11% [27, 29]. Moreover, another review and meta-analysis of depression in the Ethiopian community found that a pooled depression prevalence of 20.5% [30] which was also lower when compared to the current finding. This variance could happen because of denial of liberty due to imprisonment resulting in a denial of choices usually reserved for the outside community [67, 68]. Moreover, external factors such as overcrowded living conditions, poor quality of life, poor living environment as well as Internal factors such as a guilty feeling of the offenses committed, stigmatization [67, 69] could cause the magnitude of depression in the prison to be higher as compared to the community.

A considerable regional variation on the pooled magnitude of depression in prisoners was observed in this review and meta-analysis study. An apparent difference in the pooled prevalence of depression ranges from as low as 41.9% in the Oromia region to 72.9% in southern Ethiopia. The difference in factors related to the prison setting and administrative procedures for handling the mechanism of prisoners across the regions would be considered as possible justifications. Variation in the detection instrument used such as PHQ-9 for studies in the Amhara region, BDI-II in Oromia region and both PHQ-9 and BDI-II in southern regions might also cause such difference. Moreover, variation in cultural, economic and environmental conditions could also be responsible for such discrepancy.

Regarding the variation of the pooled magnitude of depression across measurement instruments, the average magnitude of depression as measured by Beck depression inventory-II (BDI-II) was higher (56.15%) than the average magnitude obtained when studies that assessed depression with PHQ-9 was pooled (51.25%). This might happened because studies included in the current meta-analysis and that utilized PHQ-9 depression screening instrument [20, 22, 26] incorporated a relatively larger sample size as compared to those studies which utilized beck depression inventory-II and it is statistically obvious that adequate sample size will lead to a precise and accurate result.

Moreover, the slightly lower pooled prevalence of depression in prisoners was observed in studies that utilized a sample size greater than 350 (51.93%) than the pooled magnitude of depression in prisoners that used a sample size less than 350 participants (54.13%). The reason could be a larger sample size decreases the probability of a standard error thereby providing a more precise and reliable result with strong power despite the cost to time and money.

Regarding the associated factors, a narrative review of a factor in this review illustrated that factors like health satisfaction rated as moderate, being sentenced for 1–5 years, having a plan to commit suicide, having a family history of mental illness, having a history of the previous incarceration, lack of job in the prison, lifetime alcohol use, primary level of education and perceived stigma, long stay in prison ≥10 years, being widowed, educated at college or university level and having chronic medical illness were found to had a strong and positive association with depression in Ethiopian prisoners.

### Difference between studies incorporated in the current review and meta-analysis study

This meta-analysis study was obtained to have a high degree of heterogeneity between the studies incorporated in pooling the prevalence of depression at prisoners of Ethiopia. The analysis of subgroups for detection of sources of heterogeneity was done and region of the country where the study was done, data collection tools and sample size were identified to contribute to the existing variation between the studies incorporated in the analysis. Moreover, we performed one study leave out at a time sensitivity analysis to explore further the impact of a single study on the overall estimate: whether a particular study included in the pooled effect size for depression in prisoners in Ethiopia was influential but our analysis showed that the heterogeneity between the nine included studies in the pooled prevalence was not influenced by a single particular study.

This study has limitations such as a small number of studies incorporated in the subgroup analysis that might minimize the estimate precision. Besides, the presence of heterogeneity in the pooled effect size was also the second limitation. Moreover, we describe the associated factors for prisoner’s depression only qualitatively since studies included did not report a consistent effect size for it.

## Conclusion

This review and meta-analysis study obtained a pooled magnitude of depression in prisoner’s population in Ethiopia to be very high, 53.40%(95% CI: 41.33, 65.46) and this pooled estimate was under the influence of considerable heterogeneity. Our, subgroup analysis showed that the pooled effect size for the depression prevalence was significantly in the southern region of the country than the pooled magnitude of depression in Oromia, southwest Ethiopia. Besides, the pooled prevalence of depression in Ethiopian prisoners was significantly higher as measured by the Beck Depression Inventory-II tool than the pooled prevalence obtained by the patient health questionnaire. Moreover, studies that utilized a slightly larger sample size (≥350) when pooled provided a significantly lower pooled magnitude of depression among prisoners in Ethiopia than the pooled prevalence of depression obtained when studies which utilized smaller sample size (< 350). This pooled effect size of depression in the prisoner’s population in Ethiopia obtained is very important as it showed aggregated evidence of the burden of depression in the targeted population which would be an important figure for further interventional activities in the area. Special consideration should be given to prisoners with a family history of mental illness, having a history of the previous incarceration, lack of job in the prison, lifetime alcohol use, primary level of education and perceived stigma, long stay in prison ≥10 years and having a chronic medical illness. This study would therefore be important for health care interventions for depression of prisoners and the associated factors mentioned above.

## Supplementary information

**Additional file 1.** PRISMA-P 2015 Checklist.

**Additional file 2.** Quality assessment result of the studies included in this meta-analysis.

## Data Availability

All data regarding this research work is incorporated in the paper.

## Abbreviations

BDI-II: Beck Depression Inventory
CI: Confidence Interval
ICD-10: International Classification for Diseases-10
LMICs: Low and Middle-Income Countries
OR: Odds Ratio
PHQ-9: Patient Health Questionnaire-9
PRISMA-P: Preferred Reporting Items for Systematic Reviews and Meta-analysis
WHO: World Health Organization
