# Supplementary material for: A meta-analysis of depressive symptoms among Ethiopian prisoners and a narrative description of its associated factors: a country based systematic review and meta-analysis study
Source: BMC Psychiatry. 2020 Jun 5;20:281. doi: 10.1186/s12888-020-02662-5 (PMC7275530; doi:10.1186/s12888-020-02662-5)
Supplement: Supplementary file 2 — Additional file 2. Quality assessment result of the studies included in this meta-analysis. [file 12888_2020_2662_MOESM2_ESM.docx]

**Additional file 2**: Quality assessment result of the studies included in this meta-analysis

| No | Study ID | Representation | sampling | Random selection | Non-response bias | Data collection | Case definition | Reliability and validity | Method of data collection | Prevalence period | Numerator and denominator | Summary assessment |
| --- | --- | --- | --- | --- | --- | --- | --- | --- | --- | --- | --- | --- |
| 1 | Alemayehu et al([19](#_ENREF_19)) | 1 | 1 | 1 | 0 | 1 | 1 | 0 | 1 | 1 | 1 | 8 |
| 2 | Reta et al([24](#_ENREF_24)) | 1 | 1 | 1 | 0 | 1 | 1 | 0 | 0 | 0 | 1 | 7 |
| 3 | Beyene et al([21](#_ENREF_21)) | 1 | 1 | 1 | 1 | 1 | 1 | 1 | 1 | 1 | 1 | 10 |
| 4 | Abdu et al. 2018([22](#_ENREF_22)). | 1 | 1 | 1 | 1 | 1 | 1 | 0 | 1 | 0 | 1 | 8 |
| 5 | Agegnehu et al([23](#_ENREF_23)) | 1 | 1 | 1 | 0 | 1 | 0 | 0 | 1 | 1 | 0 | 6 |
| 6 | Bedaso et al([20](#_ENREF_20)) | 1 | 1 | 1 | 0 | 1 | 1 | 0 | 1 | 1 | 1 | 8 |
| 7 | Getaneh et al([25](#_ENREF_25)) | 1 | 1 | 1 | 1 | 1 | 1 | 0 | 1 | 1 |  | 9 |
| 8 | Teferra([63](#_ENREF_63)) | 0 | 0 | 1 | 0 | 1 | 1 | 0 | 1 | 1 | 1 | 6 |
| 9 | Tirfeneh.et al([9](#_ENREF_9)) | 1 | 1 | 1 | 0 | 1 | 0 | 0 | 1 | 1 | 0 | 6 |

Yes=1, No=0

**The overall risk of bias scored based on the number of high risk of bias per study; a score of 8–10 indicating ‘low risk of bias and high quality study’, a score of 5–7 implies ‘moderate risk’ of bias and quality and 0–4: ‘high risk of bias and low quality study’.**

**Risk of bias assessment tool: Yes (low risk); No (high risk)**

1. **Representation**: Was the study population a close representation of the national population?

2. **Sampling**: Was the sampling frame a true or close representation of the target population?

3. **Random selection:** Was some form of random selection used to select the sample OR was a census undertaken?

4. **Non-response bias:** Was the likelihood of non-response bias minimal?

5. **Data collection**: Were data collected directly from the subjects?

6. **Case definition**: Was an acceptable case definition used in the study?

7. **Reliability and validity of study tool**: Was the study instrument that measured the parameter of interest show to have reliability and validity?

8**. Data collection**: Was the same mode of data collection used for all subjects?

9. **Prevalence period**: Was the length of the prevalence period for the parameter of interest appropriate?

10. **Numerators and denominators**: Were the numerator(s) and denominator(s) for the parameter of interest appropriate?
